# Reliability and validity study of the Turkish form of the family appraisal of caregiving questionnaire for palliative care (FACQ-PC)

**DOI:** 10.1017/S1478951526102053

**Published:** 2026-03-26

**Authors:** Yusuf Adnan Güçlü, Gülay Dirik, Elçin Yorulmaz, Nil Tekin, Aylin Demirci

**Affiliations:** 1 Department of Family Medicine, Health Sciences University, Izmir Faculty of Medicine, Tepecik Trainning and Research Hospital, Izmir, Türkiye; 2 Department of Psychology, Dokuz Eylül University, Izmir, Türkiye; 3 Department of Family Medicine, Health Sciences University, Tepecik Trainning and Research Hospital, Izmir, Türkiye

**Keywords:** Palliative care provision, informal caregivers, negative outcomes, positive caregiving appraisal, family well-being

## Abstract

**Objective:**

This study aimed to translate the Family Appraisal of Caregiving Questionnaire for Palliative Care (FACQ-PC) into Turkish and to examine its psychometric properties.

**Methods:**

After completing the necessary translation stages, 190 participants (109 women and 81 men) with a mean age of 43.63 years (SD = 11.83), who provided care to individuals requiring palliative care, were recruited using convenience sampling. Participants completed the Sociodemographic Information Form, FACQ-PC, Burden Interview, Positive and Negative Affect Scale, and Palliative Performance Scale. Subsequently, reliability and validity analyses were conducted on the collected data.

**Results:**

Reliability analyses included internal consistency coefficients and test–retest reliability. Cronbach’s alpha coefficients were 0.88 for the negative outcome’s subscale, 0.90 for the positive caregiving appraisal subscale, and 0.82 for the family well-being subscale. Pearson’s correlation coefficients for test–retest reliability were 0.95, 0.87, and 0.94 for the negative outcomes, positive caregiving appraisal, and family well-being subscales, respectively. Validity analyses revealed a 3-factor structure similar to that of the Polish version but different from that of the original version. Based on factor loadings, two items were removed from the scale, resulting in a final 23-item version. Examination of the factor loadings revealed that these 2 items did not load onto any factor.

**Significance of results:**

The reliability and validity analyses indicated that the Turkish version is a reliable and valid measurement tool for research and clinical applications. This tool is recommended for addressing the challenges faced by primary care physicians, health-care professionals working in home health and palliative care units, as well as family members and relatives who provide palliative care to patients.

## Introduction

Palliative care (PC) is aimed at improving quality of life among patients with chronic illnesses and their families (WHO 2020). Studies examining PC’s effect among diverse patient populations have highlighted the significant needs of caregivers (Dunbar et al. 2018; Anderson et al. 2025; Gonçalves et al. 2025). Caregiving has both positive and negative aspects (Tomita et al. 2025). For negative aspects, caregivers of patients in need of PC report psychological distress (Ullrich et al. 2025; Wu et al. 2025), anxiety (Grov et al. 2005; Mystakidou et al. 2012; Siminoff et al. 2025), and depression (Götze et al. 2016; Yan et al. 2024). Another study has reported that the education levels of caregivers were associated with depressive symptoms (Dipio et al. 2022).

Caregivers of patients requiring PC are known to experience psychological distress (Govina et al. 2019) and a high caregiving burden. Studies examining factors associated with mild and high levels of caregiving burden suggest that caring for patients all day and taking breaks from work were associated with caregiving burden. Notably, deterioration in post-care health, sleep deprivation, lack of breaks during care, limited support, limited social connections outside the home, and insufficient time for self-care also contribute to a high level of caregiving burden (Suresh et al. 2025). Studies have demonstrated the need for family-centered approaches in PC (Mehta et al. 2009; WHO 2023).

In a study of patients with cancer receiving PC at home, 60% of the participants reported positive aspects of providing care despite the challenges (Hudson 2004). When delivering PC to patients with advanced cancer, most caregivers reported a closer and more connected relationship with the patient (Song et al. 2024). Moreover, health-care professionals focusing on supporting caregivers and enhancing positive outcomes from their experiences may help caregivers cope with challenges and improve their quality of life (Song et al. 2024).

Individuals have basic assumptions about overall benevolence of the world, meaningfulness of the world, and self-worth (Janoff-Bulman 1992). According to Janoff-Bulman (1992), difficult experiences shatter individuals’ basic assumptions about the self and the world. In the aftermath of difficult experiences, these basic assumptions must be modified, and this process may lead to changes across major areas of life. Through cognitive adaptation and reconstruction of these beliefs, the impact of difficult experience may lessen, and this process can lead to positive outcomes known as posttraumatic growth (PTG). PC is also a difficult experience. Therefore, it may lead to PTG.

PTG can be defined as a process of reconstructing one’s self and life scenario that requires cognitive, emotional, and social effort, leading to a positive identity change According to Tedeschi and Calhoun PTG involves change in all or only certain domains of five subdomains. These domains are Increased Personal Strength, Recognition of New Possibilities, Greater Appreciation of Life, More Intimate Relationship with Others, and Spiritual/Existential Development (Tedeschi and Calhoun 2004).

A literature review based on this information has revealed that despite the inherent challenges, providing PC to patients is associated with several positive outcomes. Therefore, measurement tools are required to assess this process. In addition to the scarcity of scales specific to caregivers of individuals requiring PC, existing scales are broad, often evaluating multiple dimensions of caregiving. Thus, a measurement tool that can holistically evaluate caregivers’ experiences throughout the PC process is essential. However, the available evidence indicates that no scale in our country considers the individual and social effects of the caregiving process, alongside its positive and negative aspects.

This study aimed to fill this gap in the national literature by adapting and translating the Family Appraisal of Caregiving Questionnaire for Palliative Care (FACQ-PC), developed by Cooper et al. (2006) for caregivers of individuals requiring PC, into Turkish and examining its psychometric properties. In addition, this study sought to establish a Turkish version of the scale and to facilitate direct comparisons with the findings obtained from the reliability and validity study of the original scale, the Polish version, and other adaptations of the scale.

## Methods

### Participants

The target sample size for the study was set at 150, based on the number of scale items to be analyzed for Turkish reliability and validity, and the projected dropout rate. Previous reliability and validity studies have recommended a sample size of 5–10 times the total number of scale items (Gökdemir and Yılmaz 2023). While creating the sample, the following criteria were considered: age > 18 years, fluency in Turkish, being a relative caregiver of an individual requiring PC, and having provided PC to a patient for at least 1 month.

The study sample comprised caregivers of individuals receiving PC who were registered at the home health-care unit of SBÜ Tepecik Training and Research Hospital and included 190 individuals aged between 20 and 86 years (mean = 43.63 and standard deviation [SD] = 11.83) who volunteered to participate in the study. Of the caregivers, 109 (57.4%) were women and 81 (42.6%) were men. Of the caregivers, 78 (39.4%) had chronic illnesses, including osteoarthritis/arthritis (*n* = 46), hypertension (*n* = 30), thyroid disease (*n* = 11), depression (*n* = 10), cancer (*n* = 7), heart disease (*n* = 3), and chronic obstructive pulmonary disease (COPD)–asthma (*n* = 1). Table 1 presents the sociodemographic characteristics of the caregivers.

**Table 1.** Sociodemographic characteristics of the caregivers

Variables	*N*	%	Mean	SD
**Sex**				
Female	109	57.4		
Male	81	42.6		
**Age**			43.63	11.83
**Marital status**				
Married	144	75.8		
Single	32	16.8		
Divorced	14	7.4		
**Education level**				
Illiterate	2	1.1		
Primary school	19	10		
High school	85	44.7		
University	32	16.8		
Master’s degree	52	27.4		
**Employment status**				
Yes	121	63.7		
No	69	36.3		
**Income level**				
Low	45	23.7		
Medium	120	63.2		
High	25	13.2		
**Closeness with the person being cared for**				
Daughter	43	22.6		
Wife	35	18.4		
Daughter-in-law	19	10		
Son	44	23.2		
Grandchild	2	1.1		
Sibling	21	11.1		
Mother	24	12.6		
Other	2	1.1		
**Presence of another person providing support for caregiving**				
Yes	137	72.1		
None	53	27.9		
**Another person being cared for by the caregiver**				
Yes	16	8.4		
None	174	91.6		
**Maintenance period (years)**				
0–1	110	57.9		
2–5	61	32.1		
6–10	10	5.3		
11–20	9	4.7		
**Receiving training related to patient care**				
Yes	40	21.1		
No	150	78.9		

*Note*: SD = Standard deviation.

In this study, individuals receiving care were evaluated using the Palliative Performance Scale (PPS). Patients with a PPS score of ≤ 70% were considered suitable candidates for PC, as they typically require significant assistance with self-care activities and have a limited life expectancy (Anderson et al. 1996; Ho et al. 2008; Oğuz et al. 2021).

The PPS score in the study revealed that 87.36% of the patients had low functional status and required PC. The functional levels of patients reflect the extent to which family caregivers provide care to dependent patients, and consequently, their caregiver burden levels (Ho et al. 2008).

In this study, two family caregivers had a low caregiver burden (0–20%), 23 had a light caregiver burden (21–40%), and 48 had a moderate caregiver burden (41–60%). The care burden scores of the 117 family caregivers ranged from 61 to 88%. According to this result, 59% of the family caregivers had a heavy care burden. All 190 patients who received care had at least one chronic illness. These illnesses included cancer (*n* = 59), diabetes (*n* = 52), hypertension (*n* = 48), dementia/stroke (*n* = 42), heart disease (*n* = 41), COPD–asthma (*n* = 35), thyroid disease (*n* = 20), and depression (*n* = 22). Of these patients, 10 had tracheostomies, 3 had home ventilators, 36 had oxygen concentrators, 6 had percutaneous endoscopic gastrostomy, 1 had a nasogastric tube, and 16 had urinary catheters. Detailed sociodemographic and disease-related characteristics of the patients cared for by the study participants are presented in Table 2.

**Table 2.** Sociodemographic and disease-related characteristics of the patients

Variables	N	%	Mean	SD
**Sex**				
Female	110	57.9		
Male	80	42.1		
**Age (years)**			57.08	20.92
**Regular income**				
Yes	85	44.7		
None	105	55.3		
**Where she/he stays**				
Own home	85	44.7		
Caregiver’s home	61	32.1		
Rent	41	21.6		
Other	3	1.6		
**Patient’s dependency status**				
No dependency	36	189		
Semi-dependency	107	56.3		
Fully dependent	47	24.7		
**Pressure sore**				
Yes	33	17.4		
None	157	82.6		

*Note:* SD = Standard deviation.

Notably, for the test–retest evaluation, the Turkish FACQ-PC was administered to 51 caregivers of relatives requiring PC, aged between 22 and 86 years (*M* = 49.94, SD = 15.48), at 2–4-week intervals.

### Instruments

#### Sociodemographic information form

A researcher-developed sociodemographic information form was used to collect data on caregivers’ sex, age, marital status, employment, education, income, social security, and health status, as well as caregiving-related characteristics (e.g., relationship to the care recipient, caregiving duration, training, support, and allowances). Information on patients’ demographic and clinical characteristics was also obtained. The form consisted of 22 items.

#### FACQ-PC

FACQ-PC was developed by Cooper et al. (2006) to assess the positive and negative experiences of family members providing home care to relatives with cancer. The scale comprises 25 items and 4 subdimensions rated on a 1–5 scale. A study examining the scale’s psychometric properties identified 25 items and 4 subdimensions: Caregiver strain (*α* = 0.86), Positive caregiving appraisals (*α* = 0.73), Caregiver distress (*α* = 0.75), and Family well-being (*α* = 0.78). Caregiver strain includes perceived physical and emotional burdens, including feeling trapped and isolated due to the caregiver’s role. Positive caregiving appraisals encompass positive emotional benefits of caregiving, including trust, attachment, intimacy, and satisfaction. Caregiver distress includes negative emotional responses associated with caregiving, such as guilt, anxiety, and depression. The fourth subdimension (family well-being) reflects the quality of family functioning and perceived well-being. Factor analysis and construct validity indicated that the instrument was a suitable scale for assessing the experiences of family members caring for patients requiring PC.

The translation and the reliability and validity study of the Polish version were conducted by Paleczna and Zdończyk (2024), following data collection from 150 informal caregivers providing home care to patients with cancer. These individuals primarily cared for their mothers, fathers, or spouses. A confirmatory factor analysis was first performed on the Polish version of the scale; however, due to unsatisfactory fit indices, an exploratory factor analysis was conducted. Following this analysis, the scale was found to consist of three factors, accounting for 45% of the variance, unlike the original. Items that loaded onto two factors in the original scale (“caregiver distress” and “caregiver strain”) loaded onto a single factor in the Polish version, and were termed “negative outcomes” (*α* = 0.88). The other factors retained their original names: “Positive caregiving appraisals” (*α* = 0.86) and “Family well-being” (*α* = 0.80). In the Polish version of the scale, items 1, 9, and 11 were excluded from the subdimensions because of low factor loadings. In the original scale, the negative consequences for the caregivers’ subdimension of the Polish version showed a positive correlation with negative emotions and stress. Positive caregiver appraisals were positively correlated with positive affect, family well-being, and satisfaction with family life. These results indicate that the Polish version is a reliable and valid scale.

#### Burden interview

The Caregiver Burden Scale, developed by Zarit, Reever, and Bach-Peterson in 1980, was designed to assess the burden of caregivers. The scale comprises 22 items and is used to evaluate how caregiving affects an individual’s life. The scale is answered using a rating scale from never (0) to almost always (4). Scoring is based on 0–20 points for “little or no caregiver burden,” 21–40 points for “mild caregiver burden,” 41–60 points for “moderate caregiver burden,” and 61–88 points for “heavy caregiver burden” (Zarit and Zarit 1990). In the Turkish version, İnci and Erdem (2008) conducted reliability and validity studies, with an internal consistency coefficient of 0.95 and a test–retest reliability coefficient of *r* = 0.90. In this study, conducted with caregivers, the Cronbach’s alpha coefficient was 0.93.

#### Positive and Negative Affect Scale

The Positive and Negative Affect Scale (PANAS) was developed by Watson et al. (1988) to assess levels of positive and negative affect. The scale includes 10 statements representing negative emotions and 10 statements representing positive emotions. Each statement is rated on a 5-point scale from 1 to 5. The scores for both positive and negative emotions ranged from 10 to 50. Higher scores indicate stronger emotional intensity. Gençöz (2000) examined the reliability and validity of the Turkish version of the scale. Reliability coefficients were 0.83 for negative affect and 0.86 for positive affect. Data were collected from the caregivers of individuals requiring PC. Cronbach’s alpha coefficients were 0.90 for positive affect and 0.91 for negative affect.

#### Palliative Performance Scale

Health-care professionals use the PPS to assess the functional status of patients requiring PC. This scale was initially developed by Anderson et al. (1996). The PPS comprehensively assesses a patient’s ability to perform daily activities, addressing physical decline, level of consciousness, and the need for assistance. It comprises 11 categories with scores ranging from 0 to 100% in 10% increments. Notably, each score represents a specific level of functional ability and describes the extent of assistance required for self-care activities, including ambulation, activity level (self-care), evidence of illness, oral intake, and level of consciousness. Patients with PPS scores <70% are considered to require PC (Ho et al. 2008). The PPS score also predicts survival outcomes. In the reliability and validity study of the scale in Turkish conducted by Oğuz et al. (2021), the scores on the scale decreased with declines in patient functional status, indicating a greater need for assistance and higher levels of PC interventions.

### Procedure

First, the necessary permissions were obtained from the author, and the original text of the scale was requested. In the first stage, the scale was translated. Three expert physicians with advanced English-language skills and expertise in PC served as translators. The authors of this study reviewed the original version of the scale and the three translated versions to create a Turkish version. Next, the text was translated back into English by a person fluent in both languages and presented to the authors. After several corrections, the final version was agreed upon, and the Turkish version was created. Afterwards, the necessary permissions were obtained from the Non-Interventional Research Ethics Committee of X Training and Research Hospital (Decision no: 2025/04-06). Subsequently, data were obtained from caregivers via telephone or face-to-face interviews. First, the scale was administered to 20 family members caring for individuals requiring PC, and its comprehensibility was tested. Each item was asked individually to confirm that the Turkish version was well understood. No negative feedback was received from caregivers regarding their understanding of the scale. In addition, the scale items were reviewed for face validity by two PC physicians. The translation was confirmed to be understandable. Data were collected between April and September 2025.

### Statistical analyses

Data analysis was conducted using The Statistical Package for the Social Sciences (SPSS Statistics Version 20.0 from IBM, Chicago, IL, USA). First, data entry accuracy was verified. Next, Cronbach’s alpha coefficients were computed to evaluate the reliability of all the scales. Subsequently, Pearson’s correlation coefficients were calculated to assess the test–retest reliability of the FACQ-PC using data collected at 2–4-week intervals. Notably, confirmatory analyses were performed using the FACQ-PC, followed by exploratory analyses. Finally, Pearson’s correlation coefficients were computed to assess the scale’s construct validity (divergent and convergent).

## Results

### Confirmatory factor analysis

A confirmatory factor analysis was conducted to assess whether the factor structure of the FACQ-PC in the Turkish sample was consistent with that of the original study. However, because the results did not support the original structure and were consistent with the findings of the reliability and validity study of the Polish version, an exploratory factor analysis was conducted to determine the factor structure in the Turkish version, as was done in the Polish version.

First, to determine whether the dimensions of the original FACQ-PC (4-factor structure) were validated in the study sample, a confirmatory factor analysis was conducted using the relevant module of the Jamovi program (version 2.4.7). The results indicated that the goodness-of-fit indices did not support the original scale structure in this study. Notably, the root mean square error of approximation (RMSEA) was 0.13 (acceptable value ≤0.08), the standardized root mean squared residual (SRMR) was 0.14 (acceptable value should be ≤0.08), and the Comparative Fit Index (CFI) was 0.70 (acceptable value should be ≥0.90) (χ2 (269) = 1193, *p* < 0.001). The findings indicate a discrepancy between the observed covariance matrix and the dimensions implied by the original model. A similar finding was reported in a confirmatory factor analysis of the Polish version.

### Exploratory factor analysis

Given that the confirmatory factor analysis results showed that the original factor structure of the scale could not be confirmed in the study’s sample and that goodness-of-fit indices could not be obtained, an exploratory factor analysis was conducted to determine the factor structure of the Turkish version using the “oblimin” rotation method and principal axis factoring. To assess the suitability of the data for factor analysis, the Kaiser–Meyer–Olkin (KMO) measure of sampling adequacy and Bartlett’s test of sphericity were conducted. The KMO value was found to be 0.88, indicating that the sample was adequate for factor analysis. The results of Bartlett’s test of sphericity were significant (χ^2^(300) = 3160.75, *p* < 0.001). These findings indicate that the data are suitable for factor analysis. Exploratory factor analysis identified three dimensions with eigenvalues greater than one, which together explained 52.6% of the total variance.

A 3-factor solution was preferred because the scree plot supported a three-factor structure, which was also identified in the Polish version. The results showed that the factor structure of the Turkish version was very similar to that of the Polish version (Paleczna and Zdończyk 2024). Consistent with the Polish version, the items for the factors “caregiver’s distress” and “caregiver’s strain” were grouped into a single factor (Factor 1). Therefore, this factor is termed “Negative outcomes,” consistent with the Polish version. The other factors are called “Positive caregiving appraisals” and “Family well-being,” as in the original scale. The factor loadings of the scale, along with comparisons with the original and Polish versions, are presented in Table 3.

**Table 3.** Factors included in the original, Polish, and Turkish versions of the FACQ-PC and factor loadings of the Turkish form

Item	Original factor	Polish factor	Turkish factor	Factor loading (Turkish)
10- I feel depressed about caring for ███	Caregiver distress	Negative outcomes	Negative outcomes	0.89
14- As a caregiver, I feel my own health has suffered.	Caregiver strain	Negative outcomes	Negative outcomes	0.86
6- As a caregiver, I feel tired and run down.	Caregiver strain	Negative outcomes	Negative outcomes	0.84
7- As a caregiver, I feel I am losing control over my life.	Caregiver strain	Negative outcomes	Negative outcomes	0.84
12- I feel isolated and alone in caring for ███	Caregiver strain	Negative outcomes	Negative outcomes	0.80
11- I feel guilty about not being able to do more for ███	Caregiver distress	Item excluded Low factor loading	Negative outcomes	0.65
3- I am anxious about caring for ███	Caregiver distress	Negative outcomes	Negative outcomes	0.49
2- As a caregiver, I don’t have enough time for myself.	Caregiver strain	Negative outcomes	Negative outcomes	0.45
15- I have had to give up my social life to care for ███	Caregiver strain	Negative outcomes	Negative outcomes	0.36
17- I worry that I won’t be able to do enough to care for ███	Caregiver distress	Negative outcomes	Negative outcomes	0.31
8- It is a privilege to care for ███	Positive caregiving appraisals	Positive caregiving appraisals	Positive caregiving appraisals	0.84
1- I am committed to caring for ███	Positive caregiving appraisals	Item excluded Low factor loading	Positive caregiving appraisals	0.79
5- Caring for ███ is satisfying.	Positive caregiving appraisals	Positive caregiving appraisals	Positive caregiving appraisals	0.71
4- I am confident that I can handle most problems in caring for █	Positive caregiving appraisals	Positive caregiving appraisals	Positive caregiving appraisals	0.68
13- I feel useful in my relationship with ███	Positive caregiving appraisals	Positive caregiving appraisals	Positive caregiving appraisals	0.45
9- I am able to comfort █when he/she needs it.	Positive caregiving appraisals	Item excluded Low factor loading	Positive caregiving appraisals	0.36
24- Our family is able to talk about our feelings with each other.	Family well-being	Family well-being	Family well-being	0.80
22- I feel our family is closer because of caring for ███	Family well-being	Family well-being	Family well-being	0.77
21- Our family works together to solve problems.	Family well-being	Family well-being	Family well-being	0.73
16- Caring for ███ has made me feel closer to him/her.	Positive caregiving appraisals	Positive caregiving appraisals	Family well-being	0.59
20- Our family disagrees a lot about caring for ███*	Family well-being	Family well-being	Family well-being	0.58*
23- Our family avoids discussing their fears and concerns about caring for ███*	Family well-being	Family well-being	Family well-being	0.49*
25- Because of caring for ███ our family is better able to cope with change.	Family well-being	Positive care assessments	Family well-being	0.46
18- As a caregiver, I have not been able to do my job or study as well as I would like. *(If not currently working/studying circle 3)*	Caregiver strain	Family well-being	Family well-being	Item excluded
19-Caring for ███ creates financial difficulties.	Caregiver strain	Family well-being	Family well-being	Item excluded

*Note:*

* Inverse Item.

As shown in Table 3, Factor 1 (Negative outcomes) comprised 10 items (items 10, 14, 6, 7, 12, 11, 3, 2, 15, and 17). Item 17 loaded onto all 3 factors. However, it was assigned to “Negative outcomes (factor 1)” because it is semantically appropriate for the negative outcomes factor and also loaded onto that factor in the Polish version.

Factor 2 (Positive caregiving appraisals) comprised six items (items 8, 1, 5, 4, 13, and 9). Items 9 and 13 loaded onto both the first (Negative outcomes) and second (Positive caregiving appraisals) factors. Notably, item 9 loaded onto the “Positive caregiving appraisals” factor in the original scale, and item 13 loaded onto the “Positive caregiving appraisals” factor in both the original and Polish versions. Consequently, item 13 was also included in the Positive caregiving appraisals (Factor 2) factor in this study.

Factor 3 (Family well-being) comprised 7 items (items 24, 22, 21, 16, 20, 23, and 25). Items 20 and 23 were reverse-loaded, consistent with the original scale. Item 16 (Providing care to X made me feel closer to them), which was included in the Positive caregiving appraisal factor in the original scale, was loaded onto the Family well-being factor (Factor 3) in this study. This item was considered appropriate for the theoretical definition of the Family well-being factor. Items 20 and 23 were loaded onto both Factors 2 and 3. As these 2 items were included in the “Family well-being” factor in both the original and Polish versions of the scale, they were also included in this factor.

Examination of the Turkish FACQ-PC factor structure showed that two items did not load onto any factor. In studies examining the factor structures of the original and Polish versions, items 18 (As a caregiver, I cannot do my job or education as well as I would like) and 19 (Caring for x leads to financial difficulties), which fall under the caregiver strain subdimension of the original scale, loaded onto the “Family well-being” factor in this study. Because these items were not theoretically related to the concept of family well-being, they were removed when determining the factors. They were also excluded because they did not load onto any other factor.

### Reliability

Cronbach’s alpha coefficients were calculated to assess the internal consistency of the three FACQ-PC factors. The Cronbach’s alpha coefficients for the “Negative outcomes,” “Positive caregiving appraisals,” and “Family well-being” factors were 0.88, 0.90, and 0.82, respectively.

Test–retest reliability was also examined by re-administering the scale to 51 participants at 2–4-week intervals. The test–retest correlation coefficients were *r* = 0.95**, *p* < 0.01 for “Negative outcomes”; *r* = 0.87**, *p* < 0.01 for “Positive caregiving appraisals”; and *r* = 0.94**, *p* < 0.01 for “Family well-being.” Overall, the internal consistency and test–retest reliability coefficients indicated that the scale was highly reliable.

### Construct validity

The convergent and divergent validity of the FACQ-PC was examined. For convergent validity, the “Negative outcomes” factor of the FACQ-PC was expected to show a high positive correlation with the total score of the Burden Interview. As expected, a high positive correlation (*r* = 0.14; *p* < 0.05) was found between the “Negative outcomes” factor of the FACQ-PC and the total score of the Burden Interview.

For divergent validity, the “Positive caregiving appraisals” factor of the FACQ-PC was expected to show a strong negative correlation with the total score of the Burden Interview and the “Negative affect” subscale of the PANAS. Furthermore, the “Family well-being” factor of the FACQ-PC was expected to show a negative correlation with the Burden Interview.

As expected, the “Positive caregiving appraisals” factor of the FACQ-PC showed a negative correlation with the total score of Burden Interview (*r* = −0.30, *p* < 0.01) and with the negative affect subscale of the PANAS (*r* = −0.16, *p* < 0.05). Finally, the “Family well-being” factor of the FACQ-PC also correlated negatively with the Burden Interview (*r* = −0.29, *p* < 0.01). The results indicated that the FACQ-PC’s convergent and divergent validity were satisfactory. The findings are shown in Table 4.

**Table 4.** Descriptive statistics, subdimensions, and correlations with other variables of the FACQ-PC

	1	2	3	4	5	6	Mean/SD
1. Negative outcomes	1	0.58**	−0.00	0.14*	−0.08	−0.04	3.23/0.78
2. Positive caregiving appraisals		1	0.30**	−0.30**	0.02	−0.16*	3.00/0.97
3. Family well-being			1	−0.29**	0.09	−0.17*	3.43/0.42
4. Caregiver burden				1	0.08	0.32**	54.69/14.04
5. Positive affect					1	0.70**	35.27/7.19
6. Negative affect						1	33.50/7.65

*Note:* SD = standard deviation.

* *p <* 0.05.

** *p <* 0.01.

### Descriptive statistics, subdimensions, and correlations with other variables of the FACQ-PC

When the descriptive statistics of the scale were examined, the mean of the “Negative outcomes” factor was 3.23 (SD = 0.78), the mean of the “Positive caregiving appraisals” factor was 3 (SD = 0.97), while the mean of the “Family well-being” factor was 3.43 (SD = 0.42). The correlations among the three aspects of the FACQ-PC, along with their means and SDs, and correlations with other variables, are presented in Table 4.

In the Polish version, three factors were identified: negative outcomes (mean = 2.97 and SD = 0.83), positive caregiving appraisals (mean = 3.66 and SD = 0.81), and family well-being (mean = 3.59 and SD = 0.91) (Paleczna and Zdończyk 2024). When comparing the two samples on these three factors, the sample in this study reported higher negative outcomes (*t* (189) = 4.53; *p* < 0.001; 95% confidence intervals [CI], 0.15 and 0.37), lower positive caregiving appraisals (*t* (189) = −9.37; *p* < 0.001; 95% CI, −0.80 and −0.52), and lower family well-being (*t* (189) = −5.03; *p* < 0.001; 95% CI, −0.22 and −0.09) than the Polish sample (Paleczna and Zdończyk 2024).

When the correlations among the 3 factors were examined, the “Negative outcomes” factor was positively correlated with the “Positive caregiving appraisals” factor (*r* = 0.59; *p* < 0.001). The “Positive caregiving appraisals” factor was also positively correlated with “Family well-being” (*r* = 0.30; *p* < 0.001).

## Discussion

The scale was translated into Turkish, back-translated into English, and finalized by consensus. Reliability and validity analyses were then conducted in 190 participants to evaluate its psychometric properties. Reliability analyses included Cronbach’s alpha and Pearson’s correlations for test–retest reliability. Internal consistency coefficients for the subdimensions ranged from 0.82 to 0.90, and test–retest reliability coefficients ranged from 0.87 to 0.95, indicating good to excellent reliability (Nunnally and Bernstein 1994).

Confirmatory factor analysis did not support the original four-factor structure of the scale. Subsequently, an exploratory factor analysis was conducted, which supported a three-factor structure. In the original version, the factors “caregiver distress” and “caregiver strain” were combined into a single factor termed “negative outcomes,” consistent with the Polish version (Paleczna and Zdończyk 2024). This dimension captures caregiver burden, feelings of being trapped and isolated, and negative emotional responses, including anxiety, depression, and guilt experienced by caregivers of patients receiving PC. The remaining two factors were labelled “Positive caregiving appraisals” and “Family well-being,” as in the original scale (Cooper et al. 2006). The items “As a caregiver, I cannot do my job or education as well as I would like” and “Caring for … leads to financial difficulties,” originally part of the caregiver strain subdimension, loaded onto the “Family well-being” factor in this study but were not retained due to low factor loadings. Items with factor loadings below 0.30 may be removed (Tabachnick and Fidell 2001). Meanwhile, in more individualistic cultures, difficulties in work performance or financial strain may be interpreted as individual caregiving challenges; however, in Turkish culture, these experiences may be viewed as aspects of family functioning. Accordingly, different factor loadings may have emerged in this sample, in which most participants continued to work. While this is an interpretation based on cultural theory, it remains a hypothesis to be tested in future comparative studies. In addition to these items, the dimensions of positive caregiving appraisal and family well-being were retained in this study (Cooper et al. 2006). The two items that did not load onto any factor may be reconsidered in future adaptations of the scale in other languages or in studies with larger samples.

Convergent and divergent validity were examined to assess the scale’s construct validity. The Burden Interview showed a positive correlation with the negative outcomes, and a negative correlation with positive caregiving appraisals. In the original version of the scale, caregiver burden was assessed using the caregiver’s strain factor (Cooper et al. 2006). Although this factor was not present in this adaptation, caregivers’ difficulties were assessed as negative outcomes factors. Furthermore, a negative correlation was found between the Burden Interview and the “Family well-being” factor. Studies have shown that family relationships are also affected when individuals experience caregiver burden (Zhang et al. 2023; Kim et al. 2025; Suresh et al. 2025). Therefore, these results were expected. Conversely, a negative correlation was observed between the negative affect subdimension of the PANAS and the positive caregiving appraisal. In the positive caregiving appraisal subfactor, concepts such as perceived benefit, closeness, and dedication were assessed, and negative emotions were not included (Cooper et al. 2006), which is consistent with expectations.

Comparison with the Polish version, which has the same factor structure, showed that participants in this study reported higher scores for negative outcomes and lower scores for positive caregiving appraisals and family well-being. In other words, individuals caring for patients with PC in Türkiye were more negatively affected by the caregiving process. This pattern can be explained by caregivers’ support mechanisms and economic circumstances. Caregivers in Poland may receive more support from the health-care system during the process, whereas those in a better financial situation might receive professional support. Furthermore, factors such as the type of illness and PC duration might also influence this difference, consistent with previous studies (Bilgin and Özdemir 2024; Suresh et al. 2025).

The correlations among the three factors identified in this study were also examined. Positive caregiving appraisals were positively correlated with both negative outcomes and family well-being. Caregivers with higher positive caregiving appraisals are expected to experience better family functioning; that is, more positive perceptions of the caregiving process are associated with more favorable effects on families. However, the strong correlation (0.58) between Factor 1 (negative outcomes) and Factor 2 (positive caregiving appraisals) is noteworthy. Despite experiencing psychological distress or caregiving burden, some caregivers also reported positive experiences. This finding may be explained by the concept of posttraumatic growth (PTG), defined as the restructuring of an individual’s assumptions about the world following a traumatic life event, resulting in positive changes in interpersonal relationships, life philosophy, self-perception, spirituality, and awareness of new opportunities (Tedeschi 2004). Caregivers of individuals with various illnesses, including cancer (Cormio et al. 2014) and dementia (Ott et al. 2007), have also been shown to experience PTG. Individuals are more likely to experience PTG after negative events that substantially disrupt their core assumptions. In this context, the observed positive correlation between negative outcomes and positive caregiving appraisals may reflect concurrent increases in both factors. In other words, caregivers experience both negative and positive outcomes at the same time. This is consistent with the view of growth may occur even when psychological distress persists, and these experiences can happen simultaneously (Calhoun and Tedeschi 1998). Further studies are needed to examine how these factors change over time, and these findings may also inform intervention studies. In future studies, it is necessary to examine the variables related to posttraumatic growth, and to develop interventions on positive outcomes regarding caregiving process (Yorulmaz and Tekin 2019).

This scale evaluates three dimensions: Negative outcomes, Positive caregiving appraisals, and Family well-being. Its ability to assess these three outcomes simultaneously during the caregiving process offers practical clinical benefits and supports future research. This strength is further underscored by the fact that it is the first scale in the national literature to jointly evaluate the experiences of caregivers. Another strength of this study is the evaluation of the functional status of patients receiving PC using the PPS.

This adapted scale can be used in both research and clinical practice. Improved caregiving skills in PC can enable caregivers to provide care for longer periods and improve the quality of care. Consequently, reducing negative outcomes while increasing positive caregiving appraisals and family well-being can benefit caregivers. Evaluating the scale items can also help identify caregivers’ needs. Assessing these three factors using a practical 23-item scale allows them to be addressed effectively in clinical or support sessions. The application of the scale by the specified units should be encouraged, and after identifying the areas where family caregivers need more support in clinical practice, specific interventions should be planned.

Despite these strengths, the study has some limitations. First, to reach a larger number of participants, data were collected both by telephone and in person, which may have introduced variability in data collection and represents a methodological limitation. It is believed that participants are more cooperative and answer questions more sincerely in face-to-face interviews because a more informal environment is created. Differences between interviewers conducting the survey via telephone can also lead to various “interviewer effects” on the survey results (Blom and Korbmacher 2013). Second, most caregivers were women, with others being daughters, sons, or spouses of patients receiving care. The findings need to be generalized carefully in terms of gender due to the small number of male participants. Although this distribution is consistent with the literature (Paleczna and Zdończyk 2024), future studies should include a higher proportion of male participants. Nevertheless, the sample was heterogeneous in terms of employment status, support intake, and income level. In PC studies, many eligible participants refuse to participate (Paleczna and Zdończyk 2024); therefore, future studies should aim for more homogeneous samples, although participant recruitment remains challenging. Due to the difficulty in recruiting participants, individuals providing care for different illnesses in PC were included in the study. Although the functional status of individuals receiving PC was controlled in the study, the possibility of different experiences related to the illnesses constitutes another limitation of the study. In addition, as in the case of Poland, it is important to discuss the differences between averages in other country examples as well. For more detailed information, it is recommended that future research control for variables such as socioeconomic status, access to formal support, and patient functional status (PPS) when making such cross-cultural comparisons in order to better isolate cultural or systemic effects.

In conclusion, the FACQ-PC was adapted to Turkish populations and its psychometric properties were examined. Reliability and validity analyses indicated that it is a reliable and valid measurement tool for use in research and clinical practice. This scale is a suitable tool for health-care professionals, including primary care physicians, home health-care units, psychologists, nurses, and social workers in PC services, to assess the challenges experienced by family caregivers.
